# Measurements of Location-Dependent Nitric Oxide Levels on Skin Surface in relation to Acupuncture Point

**DOI:** 10.1155/2012/781460

**Published:** 2012-09-24

**Authors:** Yejin Ha, Misun Kim, Jiseon Nah, Minah Suh, Youngmi Lee

**Affiliations:** ^1^Department of Chemistry and Nano Science, Ewha Womans University, Seoul 120-750, Republic of Korea; ^2^Department of Biological Science, Sungkyunkwan University, Suwon 440-746, Republic of Korea; ^3^Samsung Advanced Institute for Health Sciences and Technology, Graduate School of Health Sciences and Technology, Sungkyunkwan University, Suwon 440-746, Republic of Korea

## Abstract

Location-dependent skin surface's partial nitric oxide pressure (pNO) is studied using highly sensitive amperometric NO microsensor with a small sensing area (diameter  = 76 **μ**m). The pNO level of LI4 (Hegu) acupuncture point is measured and compared with the pNO level of nonacupuncture point. In addition, the mapping of pNO is carried out over the left wrist skin area one- as well as two-dimensionally. Statistically higher pNO levels near the position of acupuncture points than non-acupuncture points are observed consistently, implying tight relationship between the level of NO release of skin and acupuncture points. The amperometric planar NO microsensor successfully monitors the heterogeneity of skin pNO distribution in high spatial resolution due to its advantageous features such as high sensitivity and small sensing dimension. The current study suggests the direct connection between NO and acupuncture points and possibly provides beneficial information to understand physiological roles and basis of the acupuncture points.

## 1. Introduction

Traditional eastern medicine describes meridians as physical channels which Qi flows through, connect body internal organs, and link the specified body skin locations, called acupuncture points [1]. The stimulation of acupuncture points by placing small needles is called acupuncture which is a medical treatment carried out to attain harmonious and balanced Qi flow and thus to improve health [2]. Acupuncture has been practiced as a medical treatment for over 2500 years in traditional eastern medicine. Nowadays, acupuncture treatment became to be employed more widely as a complementary and alternative therapy not only in eastern but in western countries [1]. In 1997, the National Institute of Health (NIH) published a consensus on the curative effect of acupuncture for some pain symptoms such as postoperative nausea and vomiting [3]. However, the definite efficacy and mechanism of the acupuncture action in relieving pain are still disputable. 

The distinctive biophysical features of acupuncture points and meridians, such as the high electrical conductance [4, 5] and possible relationship with connective tissue planes [6] and perivascular space [7], have been studied over the last few decades. Nonetheless, more direct scientific evidence of the physical existence as well as physiological functions of acupuncture points and meridians is still to seek particularly for humans, mainly due to the limitation of noninvasive technology.

In ancient acupuncture meridian theory, Qi has long been believed to be essential for sustaining life [8] and considered to be related with air and food intake, and inheritance [1]. Oxygen in air is definitely essential for our lives. Therefore, we supposed the possible relationship between acupuncture points and body oxygen supply and studied the heterogeneity of skin surface oxygen levels in relation to the acupuncture points [9]. The localized oxygen levels at acupuncture points and adjacent non-acupuncture points were measured using a highly sensitive electrochemical oxygen microsensor (sensing diameter = 25 *μ*m) [9]. Relatively higher oxygen levels were measured at the acupuncture points on hand skin surface, LI4 (Hegu) and PC8 (Laogong), than those at the corresponding non-acupuncture points. Our previous results provide a direct evidence for the presence of acupuncture points, which are possibly linked to the oxygenation of body. More recently, we also reported the mapping of oxygen levels in a fixed one- as well as two-dimensional areas of wrist skin surface [10]. The oxygen mapping demonstrated that the higher oxygen levels are, indeed, related with acupuncture points [10]. 

NO is known to be a signaling gas molecule mediating vasodilation and thus regulating blood flow and volume [11]. There are some previous studies on the relationship between NO and acupuncture points/meridians, reporting that acupuncture treatment increases blood flow [12] and expression of NO synthase (NOS), enzyme producing NO, is higher around skin acupuncture points and meridians of rat [13]. NO was also proposed as a prime candidate as a signaling molecule in the meridian system [14]. In addition, the quantification of NO_*x*_^−^ collected from the skin surface along meridian lines using NO collecting solutions containing NO absorbing compounds was reported [15]. However, these works include rather complicated methods (e.g., postmortem immunohistochemical analysis) which may have restrictions in future application for humans thus are not applicable in investigation of direct evidence of the NO and acupuncture point relations. Herein, we map the skin nitric oxide (NO) levels of human wrist area with a sensitive electrochemical NO microsensor. Since the present technique uses a tiny, yet high-resolution NO microsensor positioned over a fixed skin surface with a certain distance (~1 mm) to measure skin NO levels, it is advantageous towards human study of acupuncture points.

## 2. Materials and Methods

### 2.1. Electrochemical NO Microsensor

A planar amperometric microsensor highly selective for nitric oxide was prepared as described elsewhere [16]. The nitric oxide microsensor is composed of a glass-sealed Pt disc anode (Pt diameter = 76 *μ*m, Sigma Aldrich) and a coiled Ag/AgCl wire cathode (127-*μ*m diameter, Sigma Aldrich) immersed in an internal solution and covered with PTFE gas-permeable membrane (W. L. Gore & Associates, thickness <19 *μ*m, porosity 50%, pore size 0.05 *μ*m). The internal solution contained 30 mM NaCl and 0.3 mM HCl in deionized water. The surface of the Pt disc anode was electrochemically deposited with additional porous Pt layer by cyclic voltammetry carried out in 3% H_2_PtCl_6_ solution (YSI Inc., USA) to obtain the enhanced sensitivity to NO. A potential of +0.75 V (versus Ag/AgCl anode) was applied to the platinized Pt anode to induce the favorable electrochemical NO oxidation. NO oxidation current was measured between the cathode and anode, as a function of time using CHI1000A electrochemical analyzer (CH Instruments Inc., USA). For the calibration of the prepared NO microsensor, the sensor current was measured while the concentration of NO was increased in deoxygenated phosphate-buffered saline (PBS, pH 7.4, Fisher Scientific) solution by successive several injections of a given amount of NO standard solution. NO standard solution was prepared by bubbling deoxygenated PBS solution (pH 7.4) with NO gas (Dong-A Gas Co., Seoul, Republic of Korea) for 30 min. The concentrations of the standard solution was calculated as 1.91 mM using Henry's law assuming ideal dilute solutions and Henry's law constants for NO of 526.3 atom/M [17]. 

### 2.2. Skin NO Measurements

Skin NO measurements, NO mapping, were performed similarly to the skin oxygen measurements described previously [9, 10]. Briefly, as-prepared planar NO microsensor was positioned above the skin location of interest to which a drop of PBS (pH = 7.4) solution (15 *μ*L) was applied. The vertical distance between the sensor and plane and skin surface separation was maintained as ~1 mm using a micromanipulator (World Precision Instrumentation Inc., Sarasota, FL, USA). The sensor current proportional to the partial NO pressure (pNO) was monitored. Once a stable current signal was achieved, the sensor was moved and positioned over the second skin point of interest with the same vertical distance, ~1 mm, while the sensor current was monitored continuously. After the stable current was acquired at the second point, the sensor was moved to the third point to measure the pNO level at that location. This whole procedure was repeated until the measurements of pNO levels for all the projected points were completed. The measured sensor currents were converted to the corresponding pNO levels using prior calibration curves recorded before the measurements.

**Figure 1:**
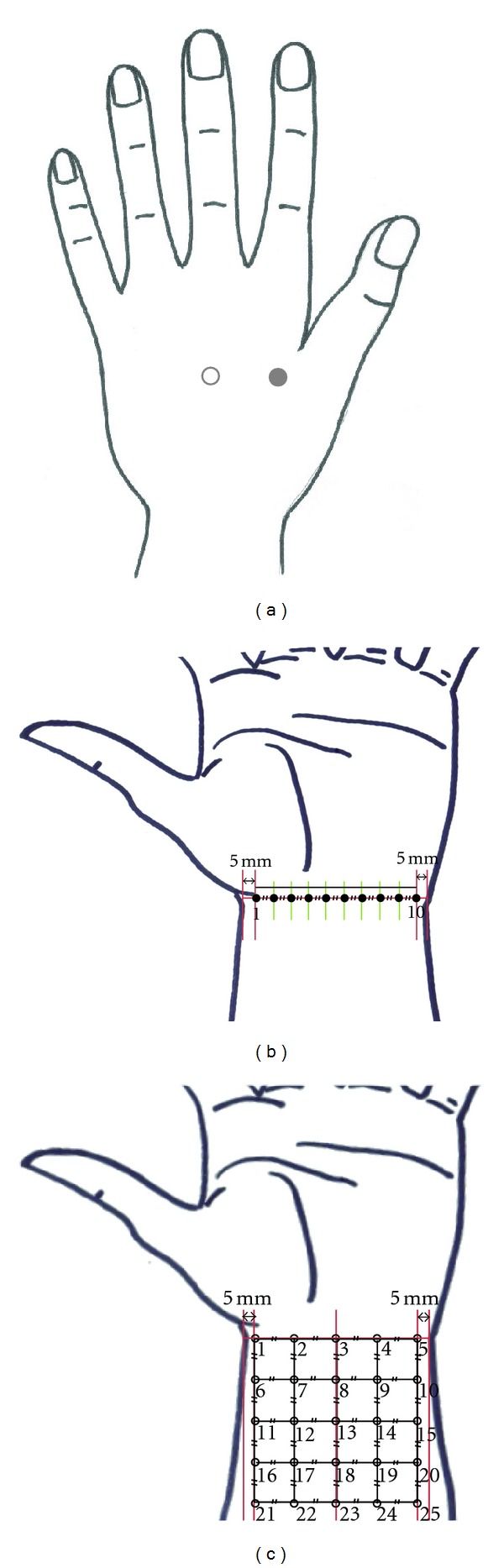
Schematic illustration for the points on the skin where a NO microsensor was positioned for pNO measurement: (a) at LI4 acupuncture point (solid circle) and non-acupuncture point (open circle); (b) 10 points along one-dimensional line on left wrist transverse crease; (c) at 25 different points within two-dimensional square area. The points, No. 1, 10 in (b) and the points, No. 1, 5 in (c) were positioned 5 mm apart from the left and right sides of the wrist. Symbol// represents the same separation.

The measurements of pNO levels were carried out in three different designs. First, pNO levels between acupuncture point and non-acupuncture point were compared. The pNO was measured over an acupuncture point (LI 4, Hegu) and then nearby non-acupuncture point ~3 cm apart with three-time repetition as shown in Figure 1(a). Second, the pNO was monitored along one-dimensional single line over left wrist in a blind fashion. The pNO levels were measured at 10 different points along the line on the anterior aspect of the left hand-wrist transverse crease. The 10 points were evenly distributed with the same separation (*d* = 4.5 − 5.5 mm depending on individual subject) between two adjacent points. The first point and the last tenth point were positioned 5 mm apart from the left and right sides of the wrist as shown in Figure 1(b). Last, the pNO measurements were performed on 25 different points equally partitioning a square area of the left wrist (Figure 1(c)). The first five points were located over the lateral line on the anterior aspect of the left hand-wrist boundary crease while the first point and the fifth point were positioned 5 mm apart from the left and right sides, respectively. 

The measurements were carried out for five healthy volunteers (average age = 23.7) in calm and restful conditions at room temperature. All the volunteers washed the projected body parts with an antibacterial hand soap, rinsed out thoroughly with water, and then fully dried before the skin pNO measurements. Any of the subjects were never treated with acupuncture needle insertion at the skin locations investigated prior to the experiments. 

### 2.3. Data Analysis and Statistics

At each point of each subject, the average of the data acquired for the last 50 s before the sensor movement to another point was taken as the pNO value corresponding to that point. This verifies that sufficiently equilibrated pNO value is measured. The averaged data for the same skin location of five different subjects were also averaged with standard deviation calculation. The data for some points showing relatively higher pNO values were compared with those at other points exhibiting relatively lower pNO values using a paired *t*-test with a Bonferroni correction. *P* value < 0.05 was considered significantly different in statistical meaning. 

## 3. Results and Discussion

**Figure 2:**
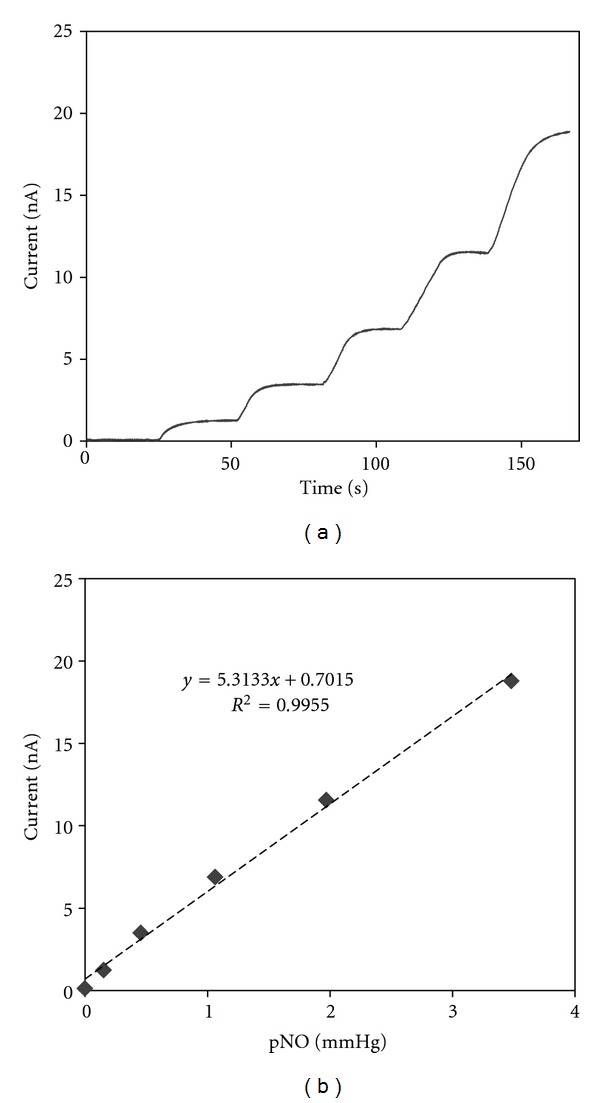
(a) A typical dynamic amperometric response curve of a NO microsensor to the varying NO concentration. (b) Corresponding calibration curve in terms of pNO.

Figure 2(a) shows the dynamic amperometric response of a NO sensor to the increased pNO in deoxygenated PBS solution. The sensor current induced by NO oxidation linearly increases as the pNO increases. High sensitivity of 5.36 ± 0.34 nA/mmHg (*n* = 5) with good linearity (*R* ^2^ > 0.99) is observed in the corresponding calibration curve (Figure 2(b)). The sensor showed no discernible responses to the addition of some biological interferents such as nitrite, indicating the selectivity to NO achieved with the aid of sensor covering PTFE gas-permeable membrane [16]. The sensor sensitivity varied within < ~5% before and after the skin pNO measurements and <0.5% for the temperature change between 25 and 35°C, confirming the sensor stability.

**Figure 3:**
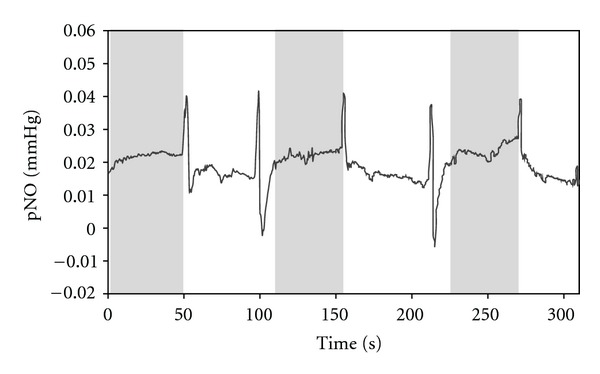
A representative pNO measurement as a function of time when the sensor was moved between LI4 acupuncture point (gray-colored) and nonacupuncture point (noncolored) indicated in Figure 1(a). The sensor end plane to skin surface distance, ~1 mm.

Figure 3 presents a typical pNO recording obtained as a function of time, while the sensor is moved between LI4 acupuncture point (gray-colored region) and non-acupuncture point (noncolored region). The measurements were repeated for three times. Sharp current peaks are noise signals caused by the sensor repositioning from one to the other points. Relatively higher pNO levels were measured over the LI4 point than non-acupuncture point consistently for all five subjects without exceptions. The absolute pNO, however, has a wide range of the values depending on subject entities presumably due to individual physiological conditions of the volunteers. Thus, for the statistics and comparison, the pNO value was normalized to the average of the pNO measured during a course of the overall measurement for each subject as follows:

(1) $$\begin{array}{c} {\text{pNO}}_{\mathrm{norm}}=\frac{\text{pNO}}{{\text{pNO}}_{\text{avg}}}, \end{array}$$

where pNO_norm⁡_ is the normalized pNO; pNO is the measured pNO value at each time point; pNO_avg_ is the average of all the pNO values measured throughout the overall experiment for each subject. Thus, the pNO_norm⁡_ values less or greater than one represent the measured absolute pNO values lower or higher than the average, respectively.

The averaged pNO_norm⁡_ for five subjects were 1.239 (±0.067) at LI4 and 0.755 (±0.060) at non-acupuncture point. The measured pNO level at LI4 point was significantly different (*P* < 0.001) compared to the corresponding non-acupuncture point. 

**Figure 4:**
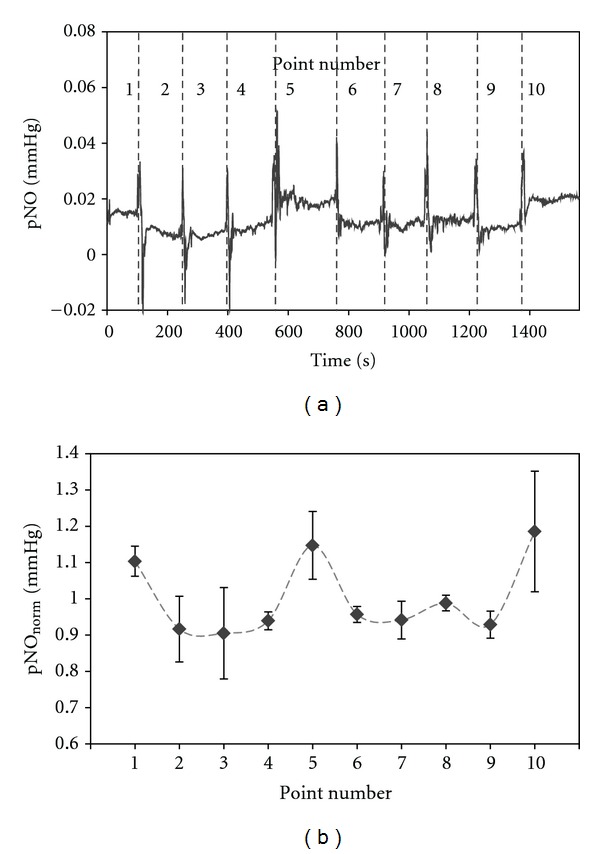
(a) A representative pNO measurement along the left wrist transverse crease as shown in Figure 1(b). The pNO values were measured continuously at 10 different points with the one-dimensional sensor movement in the direction of point No. 1 to 10. (b) Averaged pNO_norm⁡_ levels (*n* = 5) for 10 different points. The sensor's measurements at each point were averaged across five subjects. A paired *t*-test with a Bonferroni correction was conducted for eight representative points (No. 1, 2, 4, 5, 6, 7, 8, and 9), and the *P* values are summarized in Table 1.

Figure 4(a) demonstrates a representative pNO measurement at 10 different locations along the transverse wrist crease line as shown in Figure 1(b). The measurement at each location is differentiated with vertical-dashed line in Figure 4(a). Again, sharp current noise signals are observed due to the repositioning of sensor. The data clearly shows the relatively higher pNO levels at the points No. 1, 5, and 10 compared to the other parts. Similar trends of the location-dependent pNO levels were observed for all five different subjects without exceptions.  Figure 4(b) displays the pNO_norm⁡_ (normalized pNO for each subject as in (1)) averaged for five entire subjects (with standard deviation) corresponding to the indicated specific point. These statistically treated data for five different subjects also exhibit the close relation of the pNO values to the skin location. In fact, higher pNO levels were measured at the points near to the acupuncture points: there are three acupuncture points, LU9 (Taiyuan), PC7 (Daling), and HT7 (Shenmen) from left to right side along the wrist transverse crease line. Rather large standard deviations of the averaged pNO_norm⁡_ values could be ascribed to the interindividual variation such as wrist circumference and health condition. In the one-dimensional study over wrist transverse crease, the range of measured pNO values was in between 0.0243 and 0.0069 mmHg, while the greatest difference between the highest and lowest pNO was 0.0051–0.0149 mmHg depending on the individual subject. 

In addition, the pNO levels at eight representative points (No. 1, 2, 4, 5, 6, 7, 8, and 9) were compared with one another. A paired *t*-test with a Bonferroni correction verifies that the relatively higher pNO values at the points No. 1 and 5 are significantly different from the lower pNO values at the points No. 2, 4, 6, 7, 8, and 9 (Table 1). 

**Figure 5:**
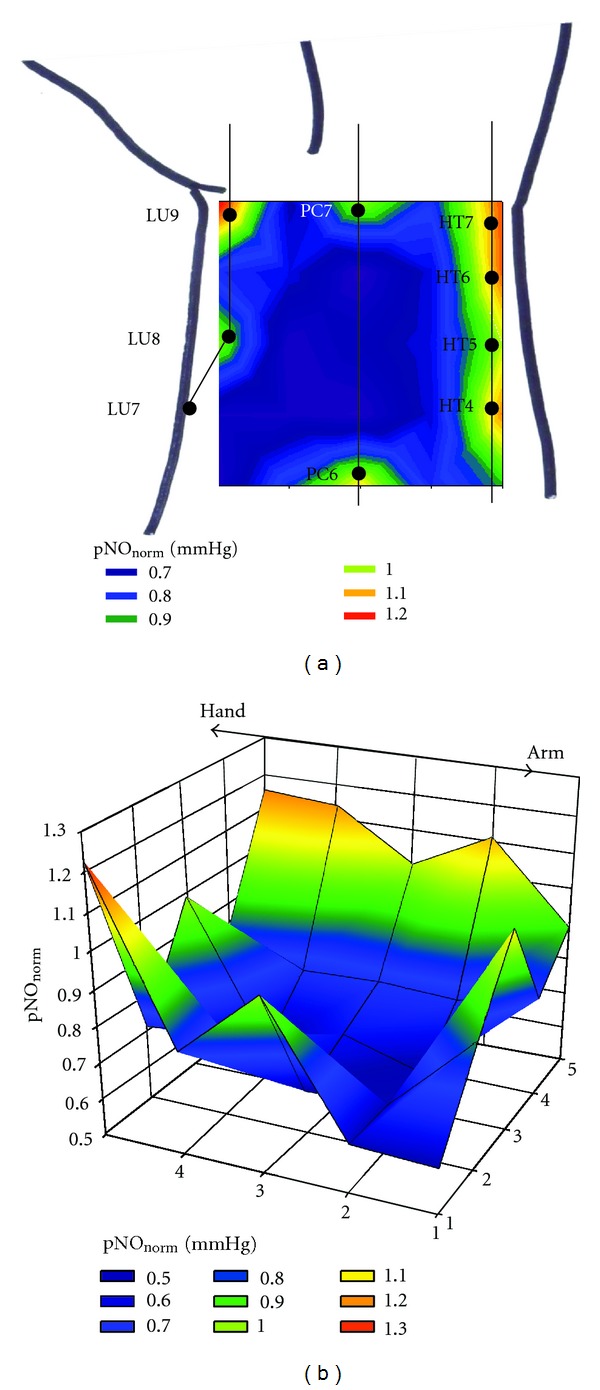
(a) 2D and (b) 3D illustrations for the color-coded contour plot of a typical example of the two-dimensional pNO measurement over the left wrist skin. A linear change in the pNO_norm⁡_ values was assumed between two adjacent points.

Figure 5 is the color-coded contour plots for a typical mapping example of pNO measurement over 25 different points in two-dimensional square area of the wrist as depicted in Figure 1(c). For these contour plots, a linear change in pNO was assumed between two adjacent points. The pNO values were varied depending on the locations, showing the heterogenous skin pNO distribution. Being in good agreement with the results shown in Figures 3 and 4, the points measured with comparatively higher pNO levels were closely related to the positions of acupuncture points. There are eight acupuncture points present in the wrist skin region where the pNO was measured: LU9 (Taiyuan) and LU8 (Jingqu) on the lung meridian; PC7 (Daling) and PC6 (Neiguan) on the pericardium meridian; HT7 (Shenmen), HT6 (Yinxi), HT5 (Tongli), and HT4 (Lingdao) on the heart meridian as indicated in Figure 5(a). Since the pNO_norm⁡_ instead of absolute pNO was used for this contour plots, the higher pNO level than the average is easily recognized by the pNO_norm⁡_ value greater than 1. In fact, the pNO_norm⁡_> 1 is found at the points closely located to the area where these acupuncture points are supposed to exist. Although the measured pNO values showed very large inter-individual variation, presumably due to the subject body size difference and physiological condition, similar pNO distribution patterns, that is, higher pNO near acupuncture points, were observed for the entire three subjects.

The observed higher pNO levels near acupuncture points in the present study are well consistent with the previous reports on higher expression of NOS enzyme around skin acupuncture points and meridians of rat [13] and higher concentration of NO metabolite along meridian lines of humans [15]. Our current work provides a clear and direct evidence of the strong relationship between high pNO and acupuncture points by *in vivo *measuring location-dependent pNO in higher spatial resolution with the aid of a highly selective tiny NO microsensor in real time. Since the NO level in atmosphere is negligible, the higher pNO over some specific skin locations suggests the emission of endogenous NO from those skin locations. In fact, the measured pNO is dependent on the sensor-to-skin surface distance, the higher pNO, the shorter sensor-skin separation is, also supporting the skin emission of NO. It should be noted that the pNO difference caused by a slight difference in the sensor-to-skin separation during the experimental course was relatively small (<0.0005 mmHg) compared to the pNO difference between acupuncture and non-acupuncture points (>0.005 mmHg), confirming the reliability of the sensor movement/reposition procedure. Importantly, the observed heterogeneous pNO distribution can be reasonably considered to be true because the pNO change caused by the sensor vertical positioning is much smaller than the location-dependent pNO difference.

There are some reports regarding the connection between gas and acupuncture points: higher pO_2_ measured in the tissue below some chosen acupuncture points of rabbit [18] and higher transcutaneous CO_2_ emission at 12 acupuncture points on the pericardium meridian of humans [19]. Our group also reported the relatively higher pO_2_ levels around acupuncture points of humans [9, 10]. Along with these previous works, the higher pNO levels near acupuncture points possibly suggest the existence of large blood vessels or main junction underneath the skin acupuncture points. Further research, however, for example, pNO analysis combined with anatomical study of vasculature, needs to be performed to clarify the possible relationship between blood vessels and acupuncture points.

## 4. Conclusions

The skin surface pNO levels as a function of location were studied using a non-invasive method with the aid of a highly sensitive NO microsensor. Both the comparison study between LI4 acupuncture and non-acupuncture points and one-, two-dimensional mapping studies for the anterior aspect of the left wrist exhibit relatively higher pNO around acupuncture points consistently with statistical significant. The amperometric NO microsensor possessed high sensitivity and small sensing dimension (diameter = 76 *μ*m) is sufficient to monitor pNO level difference depending on the skin surface location. The observed tight relationship between high skin surface pNO levels and the positions of acupuncture points provides direct and scientific evidence on the physical existence of acupuncture points and may contribute to elucidating their possible physiological/biological functions believed in eastern medicine for ages.

## Figures and Tables

**Table 1:** Calculated *P* values for the paired *t*-test (*P < 0.05).

Point number	P2	P4	P5	P6	P7	P8	P9
P1	0.0315	0.0042*	0.5010	0.0057*	0.0136*	0.0129*	0.0057*
P2		0.6975	0.0375*	0.4950	0.6994	0.2547	0.8391
P4			0.0205*	0.4066	0.9465	0.0616	0.7087
P5				0.0267*	0.0293*	0.0456*	0.02*
P6					0.6622	0.1556	0.3249
P7						0.2262	0.7472
P8							0.0765
